# Spatio-Temporal Dynamics and Diversity of Approaches in Multiscale Fire Governance

**DOI:** 10.1007/s00267-025-02365-1

**Published:** 2026-01-30

**Authors:** Christoph Neger, Octavio Romero-Cuapio, Nancy Maitrett-Bautista, Andrea Cruz-Martínez

**Affiliations:** 1 National Autonomous University of Mexico, Academic Unit of Territorial Studies Yucatán of the Institute of Geography, Mérida, Yucatán Mexico; 2 National Autonomous University of Mexico, Postgraduate Studies in Geography, Mexico City, Mexico; 3 National Autonomous University of Mexico, Faculty of Philosophy and Letters, Mexico City, Mexico

**Keywords:** Agricultural burning, Community participation, Environmental governance, Fire management, Protected area, Wildfire

## Abstract

In recent years, studies on fire governance have gained momentum, stressing that, besides technical fire management solutions, it is necessary to consider the array of stakeholders involved in this issue, including local communities. Some recent studies have suggested the need to go beyond superficial stakeholder classifications, considering nuances within stakeholder groups. The present paper adds to this discussion, highlighting the diversity of approaches, their spatial differences and temporal changes among stakeholders involved in fire governance of the La Sepultura Biosphere Reserve, a major wildfire hotspot in southern Mexico. It considers previous research alongside new information from 34 semi-structured expert interviews and fieldwork using ethnographic methods. The data are analysed within an environmental governance framework, considering qualitative social network analysis and inputs from political ecology studies. The results present a complex structure of differences in approaches and objectives that lie beyond established boundaries between stakeholder groups and classical dualities, such as between local communities and governmental agencies. The study also documents obstacles to effective stakeholder cooperation and provides some evidence on how these can be overcome. The framework developed here is relevant to other areas with similar wildfire challenges to enable a systematic revision of stakeholder roles in fire governance.

## Introduction

Conventional, suppression-focused approaches to fire management (FM) have raised widespread critiques due to their ineffectiveness and inappropriateness for fire-adapted vegetation, alongside ethical concerns regarding the restraining of fire use by smallholders, often an essential part of their livelihoods and cultures (Bilbao et al. 2019; Cammelli et al. 2019; Carmenta et al. 2021; Monzón-Alvarado 2018; Neger et al. 2024a; Schmidt et al. 2018; Tedim et al. 2020). New approaches propose to enhance FM (Bacciu et al. 2022; Martínez-Torres et al. 2018), including prescribed burning (Francos and Úbeda 2021), and risk reduction and prevention, although suppression still receives the bulk of funding (Oliveira et al. 2021; Neger et al. 2024a, b). There is also an increasing number of cases where governmental fire managers have incorporated cultural fire use into FM (Russell-Smith et al. 2017; Schmidt et al. 2018). In other cases, they at least tolerate it, although within restrictions (Cammelli et al. 2019; Monzón-Alvarado 2018).

Different concepts describe incorporating these adaptations into FM, such as the socio-ecological systems (SES) approach, the fire resilience (FR) framework, or integrated fire management (IFM). Bacciu et al. (2022) classified these terms as distinct research approaches: (1) SES as mainly addressing policies in favor of a sustainable human-fire relationship, (2) FR being interested in adaptation planning, considering climate change, and (3) IFM focusing primarily on fire ecology and technical fire use. However, Neger et al. (2024b) note that these approaches overlap, and there is no consensus on their application.

The confusion surrounding the conceptualization of FM is even greater among practitioners and political institutions; the term IFM is often lip service, whereas concrete actions remain predominantly suppression-focused (Martínez-Torres et al. 2018; Tedim et al. 2020). Thus, recent studies have recognized that environmental and socially sustainable FM does not depend solely on technical solutions and some policy adjustments; it also requires consideration of the governance structures that define FM actions.

The fire governance (FG) approach encompasses insights and applications from different approaches that converge in putting the perspectives, objectives and strategies of the whole range of involved stakeholders and their interactions centre stage as the defining aspect of FM (Devisscher et al. 2018; Monzón-Alvarado 2018; Neger et al. 2024a; Tedim et al. 2020). This approach necessarily needs to be multi-scale, related to differing understandings and management priorities at different spatial levels (Bacciu et al. 2022; Cammelli et al. 2019; Carmenta et al. 2021; Devisscher et al. 2018).

FG studies reveal the necessity to coordinate efforts and that distinct approaches and objectives may hinder cooperation. Among the main conflicts is the dichotomy between entirely fire suppression-focused and fire ecology-based approaches (Fischer and Jasny 2017; Spies et al. 2018; Spencer 2023) and misalignment in actor strategies within different spatial scales and jurisdictions (Fleming et al. 2015; Hamilton et al. 2023; Kelly et al. 2019), mostly in the United States. In addition, there are studies, primarily focused on the Global South, that deal with conflicts regarding cultural fire use, usually featuring an antagonistic relationship between local communities and governments prohibiting fire use practices and disdaining traditional knowledge (Carmenta et al. 2021; Devissher et al. 2018; Monzón-Alvarado 2018; Ponce-Calderón et al. 2020).

Usually, the stakeholder groups in these studies are clearly defined. However, there are studies showing that actual situations are often more nuanced, for example, (1) different management foci within larger organizations (Hamilton et al. 2021), (2) the questioning of the idea of “generic oppositional perspectives” of local communities versus governmental agencies (Smith et al. 2021), and (3), distinct attitudes among neighboring communities based on their economic activities and relationships with external actors (Ponce-Calderón et al. 2020) and inside communities, for instance, between genders (Weber et al. 2019), generations (Bilbao et al. 2019), between those with or without land ownership rights (Martínez-Torres et al. 2018), or generally between people with different personal experiences regarding wildfire risk (Cammelli et al. 2019).

The present study intends to delve deeper into the complexities of these stakeholder relationships and develop an approach for systematically revising stakeholder roles in FG. This detailed investigation requires a qualitative lens, considering previous literature and fieldwork applying ethnographic methods. The theoretical background for this endeavor stems from literature on the systematic analysis of environmental governance (EG) frameworks, considering governance elements (institutions, structures, and processes) alongside objectives and attributes (Bennett and Satterfield 2018; Berkes 2017; Delgado et al. 2019). For EG processes to lead to optimal outcomes, Bennett and Satterfield (2018) identify that they need to be effective – relating to aspects such as adequate capacities and leadership –, equitable and just, responsive – in terms of adaptability and flexibility–, and robust. In all these aspects, actor interrelationships are crucial, requiring well-organized coordination to be effective, genuine participation of all stakeholders to be equitable, shared learning to be adaptive, and consolidated, poly-centric connections to be robust. Within this framework, individual governance studies usually focus on specific aspects, such as adaptive capacities and related learning processes (Berkes 2017), as well as stakeholder coordination and communication, considering multi-level interrelationships (Delgado et al. 2019; Larson et al. 2018).

Whereas these insights from EG are the study’s guiding theoretical framework, it also integrates selected inputs from qualitative social network analysis (SNA) and political ecology (PE). SNA allows it to deepen the understanding of the relationships of different actors. Whereas quantitative SNA is most preoccupied with an accurate description of network structures, qualitative SNA highlights specific actor interrelationships and reconstructs the process of network formation (Glückler et al. 2017; Hollstein 2006). Moreover, it differentiates between formal and informal dynamics of stakeholder cooperation (Ruiz 2017; Spencer 2023). As in governance studies in general, the importance of different spatial levels is gaining increasing recognition in SNA (Kelly et al. 2019), as is the geographical configuration of social networks (Glückler et al. 2017). Several studies (Faas et al. 2019; Kelly et al. 2019; Spencer 2023) highlight the importance of actor collaboration in FM, due to the complexity of this issue (for instance, fires spanning multiple jurisdictions) and the limited resources of individual actors.

PE is a conceptual framework that examines power struggles and inequalities in nature-society interrelations (Robbins 2012). The present study is not a PE study per se, but advances from this field influenced it in the way authors such as Brenner and Vargas (2013) and Job and Weizenegger (2006) have defined stakeholders’ roles, interactions and conflicts and their impacts in different spatial scales, as well as their consideration of external influences. The study also integrates temporal dynamics, a prominent feature of PE that –in the case of fire-related research– has been developed by Kull (2000), Smith (2021), and Escalona and Barton (2024) and is related to the chains of explanation of environmental problems identified by early PE researchers (Blaikie and Brookfield 1987). The time scales differ widely among PE approaches (Offen 2004). The present study does not attempt to reconstruct the entire historical context in which FG in the study area developed, but examines the inherent temporal dynamics of the actions and interactions among the involved stakeholders.

The study focuses on a case study, the La Sepultura Biosphere Reserve (LSBR) in Southern Mexico, identified by previous authors as a significant wildfire hotspot (Pompa-García et al. 2018), with soaring numbers of burned areas in recent years (data provided by the National Commission of Protected Natural Areas) and documented conflicts regarding fire use between local communities and governmental agencies (Guevara-Hernández et al. 2013; Gutiérrez et al. 2017).

## Materials and Methods

### Study Area

**Fig. 1:**
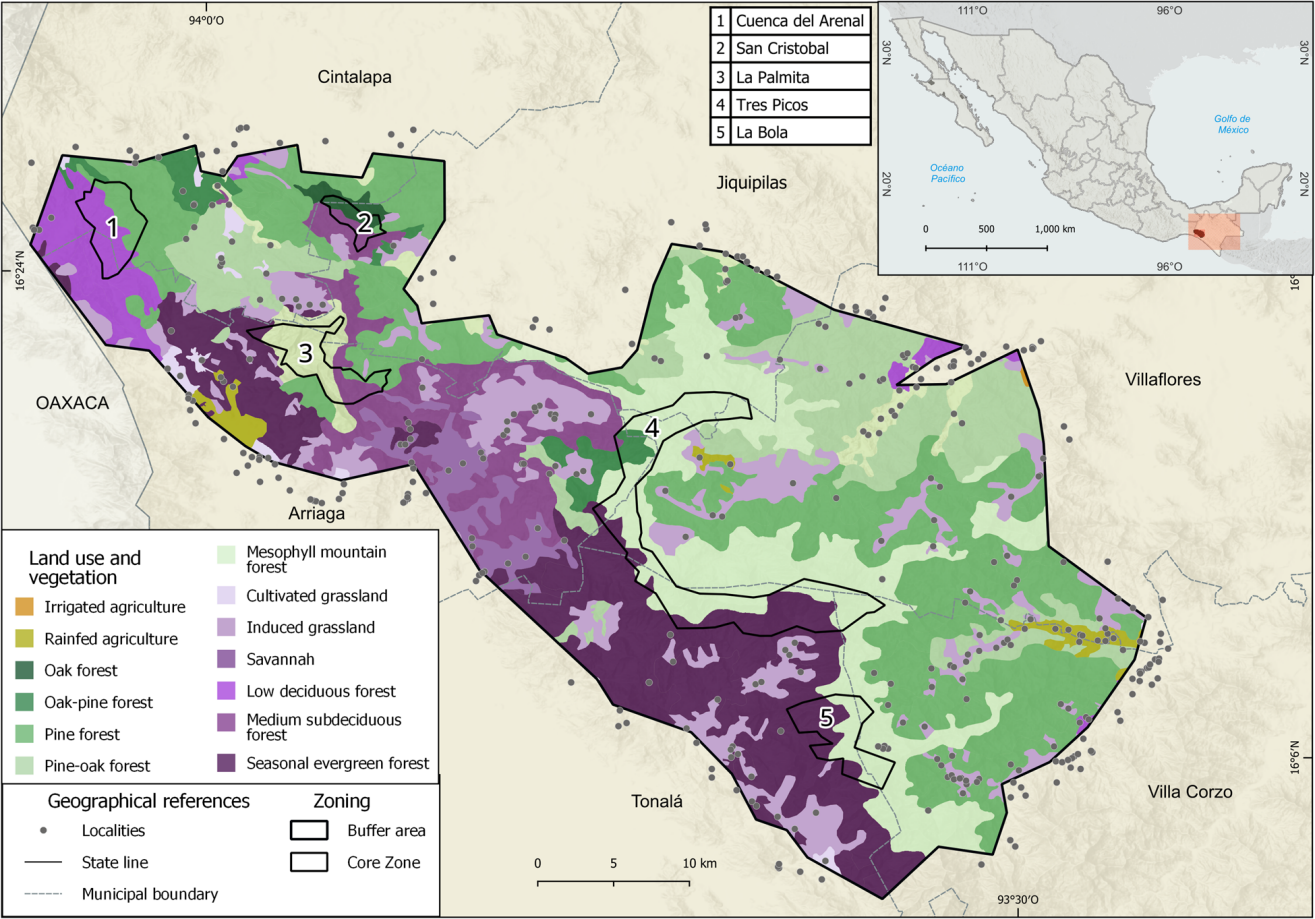
Basic cartography of the LSBR: zoning, vegetation, and population within a 5 km buffer (elaboration based on Comisión Nacional de Áreas Naturales Protegidas, 2025, and Instituto Nacional de Estadística y Geografía 2018, 2020)

The LSBR (Fig. 1), established in 1995, conserves 1,673 km2 of the Sierra Madre de Chiapas mountains (Instituto Nacional de Ecología 1999), a priority area for biodiversity conservation (Godínez-Gómez et al. 2020; Schroth et al. 2009), of national importance for water capture (Cortina-Villar et al. 2012). Furthermore, the ecosystems support local livelihoods (Braasch et al. 2017; Huffman 2010). The biosphere reserve declaration proposes integrating locals into conservation, a challenging task given the use restrictions, differing interests and discrepancies between traditional and modern practices for managing natural resources (Cortina-Villar et al. 2012).

According to the 2020 census, 9843 people live within the reserve’s boundaries, scattered among 216 small settlements, with a maximum of 988 inhabitants. Considering a buffer of 5 km, the population is 26,483 in 368 settlements with maximum 2000 inhabitants. 8.4% live in indigenous households, concentrated mainly in small villages where they make up the majority. The area shows relatively high levels of marginalization, with a literacy rate of 87.9%, compared to a national rate of 95.0% (Instituto Nacional de Estadística y Geografía 2021). Total poverty rate at the municipal level was 71.5%, including 21.7% of extreme poverty (Consejo Nacional de Evaluación de la Política Social no date). The LSBR’s population depends heavily on the primary sector, especially subsistence farming (predominantly corn and beans), coffee production and cattle ranching. Most communities are *ejidos*, governed by community assemblies (Instituto Nacional de Ecología (1999).

In recent years, the primary conservation issue in the LSBR has been fire. Burning practices by local communities seem to have a long history in the area (Gutiérrez et al. 2017). The first evidence of wildfires seen as a problem was the state government’s declaration of a high fire-risk area in the 1990s, and intense fires were registered in 1997 and 1998 (Instituto Nacional de Ecología 1999). From 2009 to 2018, the annual mean was 2428 ha of surface burned by wildfires. Since then, burned areas have increased to annually 11,651 ha from 2019 to 2024 (peaking at 27,119 ha in 2024). During these six years, La Sepultura accounted for 42.6% of burned area of federally protected areas in Chiapas and 14.8% of the state in general, according to data provided by the National Forestry Commission (CONAFOR) and he National Commission of Protected Areas (CONANP). Notably, many of these areas, with fire-adapted vegetation, present only minor fire damage and rapidly regenerate.

Overall, the area presents a complex combination of fire-dependent and fire-sensitive vegetation. It includes (1) fire-adapted forests dominated by Ocote pine *Pinus oocarpa* (up to 100% survival rate in prescribed burns), but that suffer damages from too frequent or high intensity wildfires (Rodríguez-Trejo et al. 2019; Pantoja-Campa et al. 2018); (2) oak forests adapted to low intensity wildfires (Rodríguez-Trejo et al. 2018); (3) natural and human-influenced savannahs with pine and oak trees, maintained by fires and cattle grazing (Rodríguez-Trejo et al. 2020), providing locals with a diversified income from forestry and cattle ranching (Braasch et al. 2017); (4) low tropical deciduous forests which survives wildfires, albeit with a reduced plant species diversity (Rodríguez et al. 2019); and (5) cloud forests, highly sensitive to fire, located in the highest parts of the mountains (Braasch et al. 2017; Rodríguez-Trejo 2014). According to CONAFOR data, fires between 2015 and 2024 had the following likely causes: agriculture and cattle ranching (82.3%), hunting (5.4%), other illegal activities (vandalism and conflicts, 5.0%), natural causes (lightning, 0.6%), other causes (1.7%), and unknown (5.0%).

### Data Collection and Analysis

The research reviewed previous case studies on social aspects of fire (Barrios-Calderón and Escolar-Flores 2020; Guevara-Hernández et al. 2013; Gutiérrez et al. 2017; Huffman 2010) and on fire ecology in the reserve and its surroundings (Braasch et al. 2017; Pantoja-Campa et al. 2018; Rodríguez-Trejo et al. 2018, 2020) that mentioned FM activities. The study received general data on fire incidence (2009 to 2024) from CONANP and CONAFOR, serving as supporting information to understand FM in the area. Except the use of these quantitative data, the research followed a typical qualitative research design (Creswell 2021) consisting of data collection, the thematic analysis of the data, and the presentation of the results in the form of a systematic narrative.

The central part of the research consisted of three field visits (2021, 2022, and 2023), with the application of 34 semi-structured interviews (according to the definition of Dunn 2010) with representatives of different actor groups, including five follow-up interviews in different years. The selection of interviewees followed guidelines for achieving theoretical saturation and salience (Weller et al. 2018). Some of these were group interviews, with a total of 38 participants (7 federal government, 8 state government, 3 municipal governments, 2 non-governmental organizations, 8 community representatives, 5 professional wildfire brigades contracted by CONAFOR, and 5 community wildfire brigades). The selection focused on individuals in key positions for FG. For communities, the selection included those with the highest fire incidence, those with government-sponsored fire brigades, and those featured in previous studies.

While this narrative approach was the primary source, it was combined with observational methods related to ethnographic research (Gubrium & Holstein 1999), including participatory observation documented in a research diary (as described by Seim 2021), comprising two patrols by firefighters to detect and verify potential wildfires, a capacity building workshop, and the suppression of three fires, including FM tasks such as the patrolling of natural fire lines and the opening of a fire break; the observation also included strategic discussions before starting to fight the fires, transport together with firefighters, waiting at gathering places, and sharing a meal, which allowed it to get into informal but rich conversations. Furthermore, the research included about 50 short, informal conversations with locals in public areas of two of the most populated towns of the area: Tiltepec and Tierra y Libertad (municipality Jiquipilas), to obtain an idea of the general view of FG among the local population. The fieldwork was mainly carried out by two of the authors (CN, OR). In one instance, the other two authors (NMB, ACM) joined, participating in interviews and especially in conversations in Tiltepec and Tierra y Libertad.

The research followed the ethical guidelines of the National Autonomous University of Mexico’s code of ethics, including those related to privacy and the protection of personal information. In this sense, the authors explained the study’s purpose and nature and obtained the informants’ oral consent, documented in the audio recordings. We opted to anonymize the data, due to our work on low-level conflicts and discord, where naming specific informants might not be conducive to future cooperation or might carry negative repercussions. We tried to best reflect the views of the informants, through deep, long conversations and reiterative questioning on specific key aspects, to avoid misunderstandings.

All resulting texts were coded with QDA Miner Lite software and integrated into tables and graphs for systematic interpretation. In line with the observation of Saldaña (2021) of inductive and deductive coding as a dialectical procedure, the coding process combined both these approaches, starting from predefined codes, adding additional codes based on the literature and expanding the coding during the process, adding new aspects not identified before; it also included re-reading of previously coded texts in the light of newly aggregated codes.

The description of the results followed a systematic order based on previous analysis frameworks from EG, SNA, and PE studies and the coding process. This included identifying actor roles and relationships within the area’s governance network, the temporal dimension, the attention to different spatial levels, and the graphic visualization of these aspects, following the advances of previous qualitative studies on actor relationships in environmental governance (Brenner & Vargas 2013; Martínez-Torres et al. 2018). The results reveal the different actor groups’ perception of FG. They should not be seen as hard facts, although several themes showed widespread consensus, presented here by speaking in general terms; we also point out where opinions differed. The figures and overall findings represent a synthesis most interviewees’ views, supported by the authors’ field experience and previous literature.

## Results

Six essential, interrelated aspects define FG in the study area: (1) temporal dynamics, with changes that affect the other five aspects; (2) actors and their objectives regarding FM; (3) actor interactions, classified broadly into conflict and cooperation; (4) main challenges to FM; (5) external socio-political and biophysical influences; and (6) regional disparities.

### Temporal Dynamics

One of the most striking realizations of this research was the highly dynamic nature of FG. Several phases have led to the present configuration of FM activities in the area:

In *Phase 1 (pre-1998)*, documented by previous research (Gutiérrez et al. 2017) and also by several interviews with both governmental and community actors, local communities used fire in two very distinct ways: either in a controlled manner to maintain fields and pastureland, applying cultural fire knowledge, or without any apparent care and control measures, to extend the agricultural frontier (the mentioned authors cite locals with the phrase “may the sea put it [the fire] out”, Gutiérrez et al. 2017:44), mainly without any direct external involvement.

*Phase 2 (1998 to 2000s)* brought the introduction of formalized FM in many parts of Mexico, including the recently established LSBR, following the extreme 1998 wildfire season, aided for a limited time by actors such as the US Forest Service, the international NGO The Nature Conservancy, and the national NGO Pronatura. Both federal and state environmental agencies began developing plans for FM, assigning specific personnel, and gradually establishing inter-institutional cooperation; at the beginning, fire suppression approaches and oppression of agricultural burning prevailed in the region. However, the external actors sought to involve local communities, initially in a rather top-down manner, with varying degrees of success and accompanied by conflicts, as outlined in the subsequent sections. Several stakeholders from state and federal environmental agencies slowly started adopting an IFM approach, as already noted by Gutiérrez et al. (2017), through contact with foreign actors and their own experience. The phase coincided with the introduction of the Official Mexican Norm on fire use (in short NOM-015, promulgated in 1997), which regulates permits for agricultural and technical burning.

**Fig. 2:**
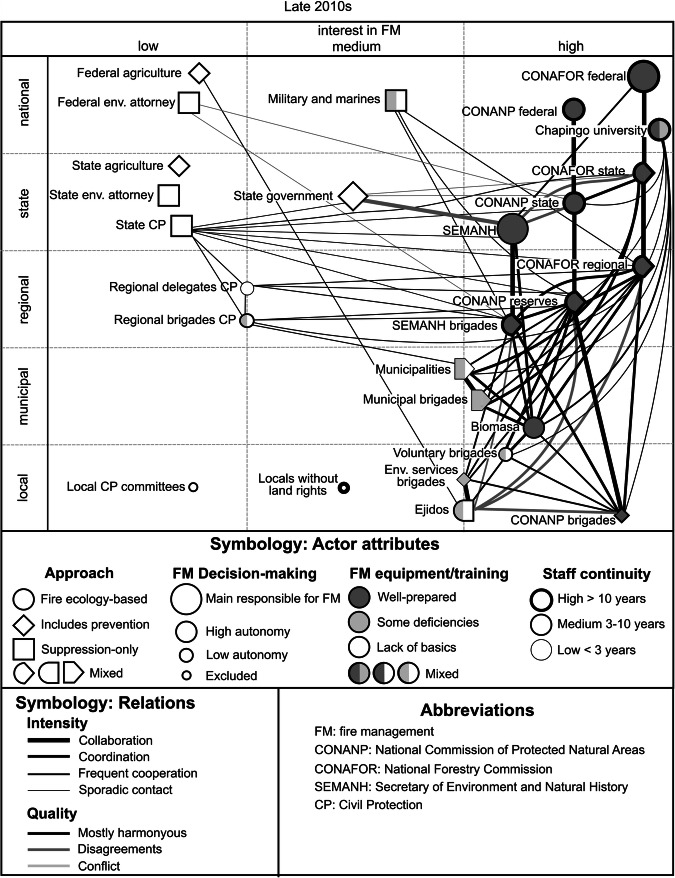
Actors and interrelationships regarding FM in the LBSR in the late 2010s (Phase 3) (elaboration based on observation in the field and the prevailing opinions of 38 interviewees from different actor groups)

During *Phase 3 (the 2010s)* (Fig. 2), the area established a relatively organized management approach based on the coordination of an operational group at the level of the state of Chiapas and regional FM centres separated into three regions, Valles Zoque, Istmo-Costa, and Frailescana, and a primarily harmonious relationship between local communities and the governmental agencies, confirmed by actors from both groups.

**Fig. 3:**
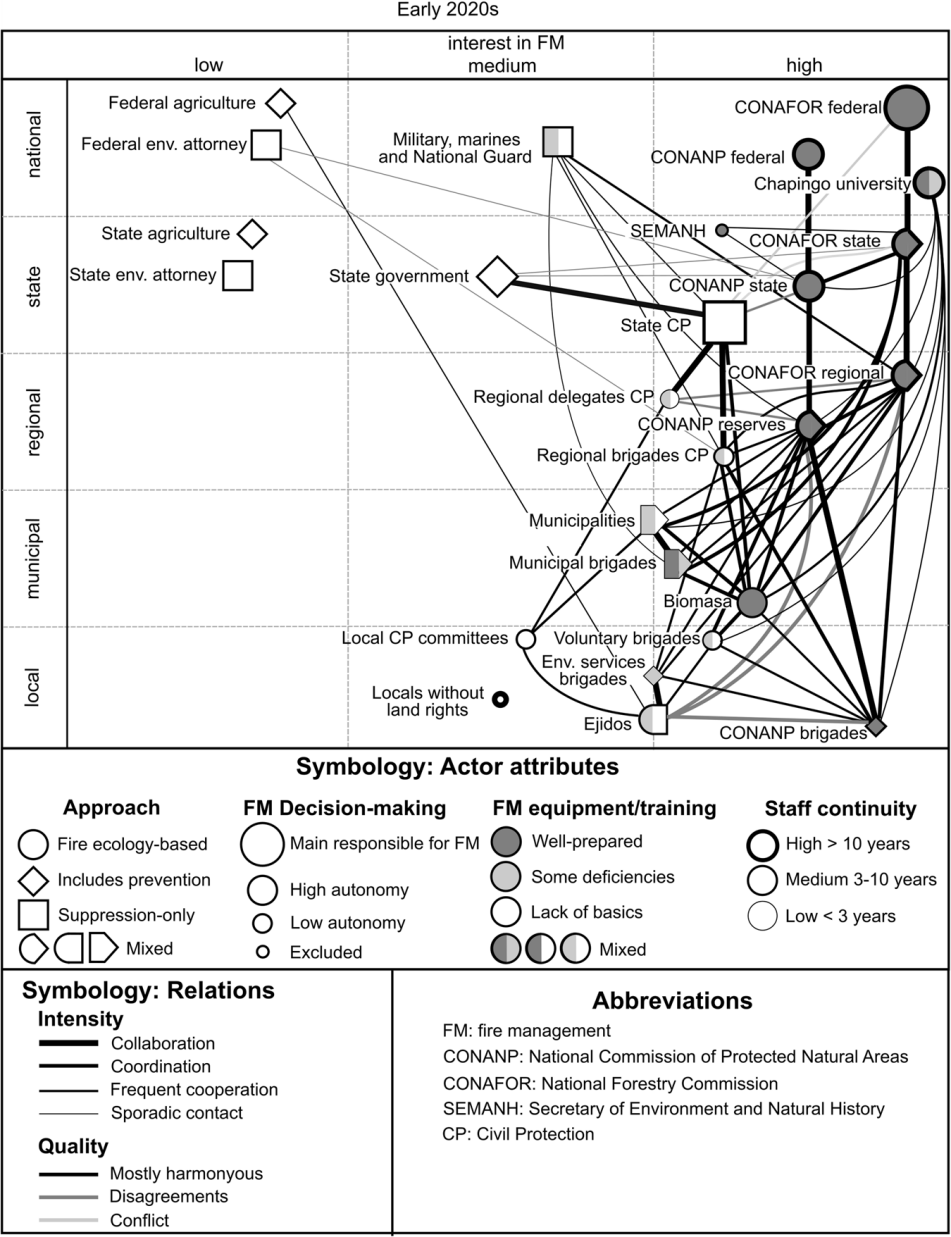
Actors and interrelationships regarding FM in the LSBR in 2020/2021 (Phase 4) (elaboration based on observation in the field and the prevailing opinions of 38 interviewees from different actor groups)

*Phase 4 (2020-2022)* (Fig. 3), besides minor effects of the COVID-19 pandemic, brought considerable disruption regarding FG, with main responsibilities assigned to the previously scarcely involved state government’s civil protection (CP) department According to a wide range of interviews at different levels and as observed in the field, this change generated conflicts and affected cooperation.

*Phase 5 (since 2023)* witnessed a relative consolidation of this new structure, with renewed cooperation and less conflict. However, burned areas reached an unprecedented high.

Phases 1 and 2 were mainly reconstructed from literature and memories of some of the leading actors, limiting access to detailed information. The best documented phases were 3 and 4, based on fieldwork, enabling the drawing of actor interactions models, shown in Figs. 2 and 3. The identification of phase 5 considered information obtained during the last fieldwork, but was not complete enough for a full representation.

### Actors and Objectives at Different Spatial Scales

The category of *governmental agencies* includes a wide array of actor groups. The *armed forces* (the Marines, the Military, and, recently, the National Guard) participate exclusively in suppressing large fires. They usually lack specialized equipment and specific FM training. In recent years, CONAFOR promoted capacity building among soldiers; however, given the military personnel’s quick rotation, these efforts have had limited success, as observed in the fieldwork.

*Federal and state environmental agencies* are at the forefront of FM, especially the CONANP, which administers the LSBR and supports several community brigades, and the CONAFOR, the main responsible for training, supporting communities with payments for environmental services and promoting the NOM-015; furthermore, they have centres with professional wildland firefighters in each of the three regions. Both have maintained strong continuity in personnel (usually, under new governments, incumbent officials change, but the FM professionals stay), institutional structures (despite the budget cuts mentioned below), and overall guidelines regarding natural resource management spanning changing federal administrations. Regarding FM, both organizations have transitioned from a suppression-focused, prohibitive agenda to a more integrated, open approach, establishing close cooperation with several communities. This conversion has not been uniform, happening first within the CONANP; some professionals at regional and state levels still maintain a reserved stance, particularly regarding cultural fire use. The situation resembles that of the State Secretary of Environment and Natural History (SEMANH) prior to 2018, before the new state government diminished the secretaries’ responsibilities as lead state actor, and completely removed its FM attributions in 2020.

*Federal and state environmental attorneys* are responsible for investigating environmental offenses, including arson in forest areas. In the early 2000s, federal authorities issued fines and even jailed some locals for agricultural burning-related accidents, leading to asymmetric conflicts between the government and local communities, as documented by Guevara-Hernández et al. (2013). However, in recent years, several other governmental actors have complained about the attorneys’ inactivity and lack of interest, leading to complete impunity of documented arson cases. The only exception was sporadic participation in legal prevention patrols alongside other actors.

By law, *federal and state agricultural agencies* should participate in implementing the NOM-015 on safe fire use. In practice, they have no personnel and programs assigned to or concrete actions regarding FM in the area. The only exception is the National Research Institute for Forestry, Agriculture and Livestock (INIFAP), which works closely with the local community Tiltepec (Jiquipilas) on agricultural programs, influencing the adoption of a zero-fire policy by the community authorities.

The state *Secretary of Civil Protection* (state CP), present in the region with regional delegates and brigades, before 2000 participated sporadically, mainly providing helicopters to reach otherwise inaccessible areas. In phase 4, due to changes in the state government in office since 2018, it was designated as the leading actor for FM coordination. According to several other governmental actors, they lacked adequate know-how and equipment and refused to receive training and counseling from the environmental agencies. Another critical issue is the frequent rotation of regional delegates (up to several times a year) observed during fieldwork, complicating medium- and long-term planning. Initially, the secretary’s posture was entirely suppression-focused and anti-fire, except for a few officials with prior experience in this field or who had moved from other agencies to CP. Eventually, more agency members went through a learning process, leading to a slightly more integrated approach in phase 5.

The *municipal governments* are a highly diverse category, characterized by frequent rotation of personnel (usually, the departments in charge are those dedicated to CP and environment or agriculture) and different approaches in each of the six municipalities that share parts of the LSBR: Cintalapa, Jiquipilas, Villaflores, Villa Corzo, Tonalá, and Arriaga. While the latter two are usually less dedicated due to lower wildfire incidence, the other four generally assign some of their resources to FM, finance their own *municipal brigades*, and manage brigades financed by CONAFOR. While municipal involvement in fire suppression is relatively high compared to many other areas of Mexico, preventive activities at the municipal level are scarce, besides participation in cultural prevention events organized by CONAFOR and CONANP. According to NOM-015, municipalities should implement burning permits, but according to the interviewees’ general experience, this has occurred only in a few exceptional cases.

The second outstanding actors, aside from governmental agencies, were *local communities*. Almost all of them had originally used fire to burn stubble on fields and to renew grasslands, based on local knowledge developed over generations (although some communities have migrated here from other areas in Chiapas and had to adapt their practices to the local environment), and usually with mutual assistance by people from the same community. Furthermore, cultural FM involves the empirical management of low-intensity wildfires in savannahs and pine forests by some local communities to obtain optimal fire benefits, documented by Pantoja-Campa et al. (2018:3, own translation): “Here a conducted wildfire is considered a wildfire which occurred under conditions which implicated a low to moderate intensity, which is why the *ejidatarios* decided to let it advance, like a controlled burn, with the objective to reduce forest fuels and regenerate the tree cover and the pasture. They limited themselves to following it and observing it, ready to proceed to control it in case the conditions would change (…)”.

This kind of knowledge is declining, as is fire use in general. On the contrary, there are also cases of more indiscriminate fire use in some villages, according to some governmental and community representatives. The community assemblies frequently define internal fire-use rules that are generally respected, with some exceptions. Some communities have proclaimed total fire bans, whereas cultural FM is still more prevalent in Indigenous communities. A changing number (declining in recent years) of communities in the reserve receive payments for environmental services managed by CONAFOR, which implies the obligation to set up *community brigades* and realize physical fire prevention. Other communities have brigades managed by CONANP or the municipality of Villaflores; in some cases, locals volunteer for fire suppression.

Another interaction between communities and the government in recent years (since phase 3) has been the establishment of local CP committees. Due to changes in responsibilities at the state level in phase 4, they were then also considered relevant for FM. At the CP central office in the state capital, these committees were presented as an effective solution for local preparedness. However, they were hardly mentioned by the secretary’s regional delegates, and other governmental actors criticized the committee’s objective as primarily political campaigning and lacking relevance for firefighting, an impression confirmed by field observations.

The interviews with community representatives revealed that those inside the LSBR and who use forest resources, such as pine resins, are keen to conserve forests, as outlined in a community representative’s remarks: “Now we are in a resin cooperative here in the *ejido* (…) and now it is my priority employment. I have another activity, which is corn and beans, but it is minimal (…). Since 2015 to this date, thank God, there has been no wildfire. Everything is controlled, at least for now. Well, we are *resineros* (resin cultivators), we protect our forest”. In contrast, interest in agriculture-based communities at the reserve’s borders is lower. There are also stark differences in opinions within communities, including those of *avencidados*, local inhabitants without land and rights to vote in the *ejido* assemblies. As observed in conversations with locals the communities Tierra y Libertad and Tiltepec, they often hold anti-fire opinions, experiencing only adverse effects (fire smoke, water shortages attributed to forest degradation) but none of the benefits. Overall, concerns regarding adverse effects of wildfire and the need for an effective control of fire use seem to be widely based on personal experiences (damages to forest resources and crops, smoke). However, outright anti-fire sentiments at the local level are generally the product of external influence, as is reflected, most notably, in the total fire ban in the community Tiltepec, reflected in the words of a local firefighter: “We in this community have this zero fire rule, it is for the wellbeing of the community, right, because by burning, to begin with, they harm the environment (…) some farmers still do it, but they shouldn’t, due to the community rule, because this causes the shortage of water.”

Overall, social capital of communities regarding fire depends primarily on (1) local management capacities related to equipment and both cultural and technical knowledge, and (2) internal cohesion and respect for the community assemblies’ rules (usually they deal with indications for controlled agricultural burning, although in one case of a resin production community, local rules also considered prescribed burning). Regarding equipment there are striking differences between volunteers who use just their simple agricultural equipment such as machetes, brigades who sporadically have received low-quality equipment from different governmental agencies (for instance, one community representative asked the interviewer: “Tell them, why don’t they give us more. Shoes, good shoes. Our shoes are crappy (*chafa*), well, they don’t last”), and communities that receive constant support from CONANP or CONAFOR. Technical knowledge depends on the communities’ contacts with external actors and on community members’ involvement in professional fire brigades.

Regarding the loss of cultural knowledge and lack of internal cohesion, the situation is generally more problematic in larger communities at the reserve’s outskirts, and less critical in small communities located close to the core zones. Also, communities outside the LSBR are influenced by a variety of governmental actors with conflicting perspectives on FM. In contrast, those within cooperate predominantly with environmental agencies. The results were inconclusive regarding the functioning of local governance structures; overall, interviewees reported that people tend to respect local FM rules, which are usually the result of agreements made by the community assemblies; however, some complained about a few exceptions of young community members using fire carelessly or of fires caused by other neighboring communities.

*Other stakeholders* include non-governmental organizations and universities. Researchers from the Chapingo Autonomous University in Texcoco, State of Mexico, have been working on studies on fire ecology in the area, on some occasions involving experimental prescribed burns, in coordination with an array of actors, including a local NGO (Biomasa), and have provided some of their insights to local fire managers. Other research in the area has had no apparent impact on local FM practices.

As outlined in the previous paragraphs, the roles and interactions of the actors have changed throughout the different phases, which is also visible in Figs. 2 and 3. The following lines resume the most important changes observed between phases 3 and 4 apparent in the figures: Regarding actor roles, the most striking change was the transition of the state secretary of CP from being listed among the actors with low interest in FM to becoming the leading actor, and related to this the disempowerment of the state environmental secretary; both changes altered the actor relationships; CP already had held ties with many other actors before, but these got stronger now and, in some cases, more conflictive. The changes at the state level had further repercussions at lower levels, with the increased relevance of regional delegates and state CP brigades, and the additional attributions of local CP committees. Municipal brigades increased their capacities, partly due to the advancement of capacity-building programs and partly by contracting personnel laid off in the state environmental secretary. Moreover, the figure shows the strengthening of the cooperation between CONAFOR and the military.

### Actor Relationships: Cooperation and Conflict

Regarding *cooperation*, there is no evidence in the FM context in the area prior to the 1998 fires, except for inter-community collaboration in conducting controlled burns. The large 1998 fires prompted spontaneous cooperation among diverse actors across the country for fire suppression, including La Sepultura. Later, little by little, actors started to align their FM efforts. At the regional level, 2005 marked the beginning of a formal IFM program for the reserve’s administration, CONAFOR, municipalities, and communities, funded by The Nature Conservancy. Later, this formal program stopped for lack of continued financing, but the same actors kept working in a coordinated manner, further including the state government’s environmental agency. Overall, the cooperation between local communities and the central responsible governmental institutions nowadays can be seen as relatively harmonious, as one firefighter contracted by the CONAFOR told: “Fortunately, we have this good coordination with the *ejidos*, there is communication, if there is a wildfire, the people assist. We just went to a fire over there in Villa Corzo and the people responded very well (…) it is not in all municipalities and in all *ejidos*, but generally we receive help from the people”.

However, as Huffman (2010:25-26) observed, there is a “delicate balance of power, cooperation and subtle resistance that producers and reserve managers constantly negotiate through daily actions and participation in government-sponsored programs”. As governmental, NGO, and community representatives alike observe, this balance is challenged by cuts to environmental programs and by unaligned policies across different governmental agencies. Factors contributing to successful cooperation are the longstanding experience and contacts of many of the involved persons, and the openness towards flexible, locally adapted solutions, for instance, regarding the application of the NOM-015.

A specific, strong cooperation has been established since the late 2010s for the realization of experimental prescribed burning efforts, under the auspices of the Chapingo Autonomous University, and with the participation of the NGO Biomasa, CONAFOR firefighters, municipal and community brigades, as well as the LSBR’s administration, supported by the Mexican Fund for Environmental Conservation. However, to date, these practices have been limited to small experimental patches, with none of the stakeholders being willing or able to provide resources for a larger-scale prescribed burning program, despite the declarations of NGO and governmental representatives that there was an urgent need in the reserve for more effective fuel management.

At the state level, a technical operative group has been set up to institutionalize cooperation in fire suppression, capacity-building and, to a lesser extent, fire prevention efforts, comprising CONANP, CONAFOR and state government representatives. In each region there are regional FM centres, including three in the LSBR area, further involving the representatives of the municipalities. Due to the discontinuity of municipal personnel, maintaining this cooperation is an ongoing struggle.

*Conflicts and disagreements* were a crucial issue in the first years of the LSBR’s existence, when it faced limited acceptance from local communities due to the lack of a genuine participatory process to establish the reserve. The start of organized FM activities was also not without different degrees of conflict at the local level. One of the most critical examples reported by Guevara-Hernández et al. (2013) involved the issuing of a fine by the Federal Environmental Attorney following a report from CONANP to a community as a whole for supposedly being responsible for having started a wildfire; some time after, two young men in the community were put into jail for some time because of an accidentally caused fire. In the first case, municipal authorities served as mediators between the community and federal agencies.

In contrast to the direct conflicts between communities and authorities, the relationships later became more complex, with lines of conflict extending across previously defined blocks. Within communities, there are varying degrees of tolerance for fire, and generally the community as a whole sanctions indiscriminate fire use, leading to intra-community conflicts with those who use fire without necessary control measures, and sometimes even against those who use fire in a controlled manner. There are also issues between communities, with accusations against neighboring villages for causing fires that spread beyond community limits, as reflected in the following statement of a community representative: “They may cause a fire from another place. I tell you, last year they affected us. Over there, they affected us (…). We had a meeting with them where we told them that it can’t be that every year, they affect us. They said, well, it wasn’t us. But then, where did the fire come from?”

At the same time, as some authorities, especially the main actors in the environmental sector, adopted an IFM approach, they came into opposition with other actors, most notably several former state governors who issued temporary fire-use bans, including prescribed burning. The implementation of these bans was limited in practice, but it contributed to distrust between communities and government actors. The situation escalated in phase 4, following the removal of the state environmental secretary from FM activities and the transfer of principal responsibility to state CP, who lacked training and know-how in FM and pursued an anti-fire policy. This led to conflicts, with a total break-up of communication in some cases, and problems in cooperation in others. According to some interviewees, this led directly to a wider spread of some fires (due to incomplete fire breaks caused by disorganization or lack of know-how) and, on one occasion, to an accident involving three persons suffering severe burns. The degree of conflict varied across the region, with most problems in the northwestern part of the reserve. One representative of a governmental institution summed the situation up like this: “And this change provoked a certain friction between the institutions and has affected the issue of cooperation. In which way? In not sending brigades or if they are combatting, in putting them under much risk to put out the fire”.

Phase 5 brought about a relaxation of this situation, as was clearly observed during the last field visit, especially among local actors, with enhanced cooperation in firefighting and with CP officials adopting a more open position toward IFM (In the words of the representative of another governmental institution: “Previously, civil protection issued a zero-fire decree. Zero fires in the state; it is not possible to burn, anyone who does it goes to jail. And what did the farmer do? Well, with this fear, he went, set fire, and went to his home. Who burned it? Who knows. But now civil protection has joined the issue of the official Mexican norm, so that people can use fire responsibly”). The main remaining point of conflict was the reporting of fire numbers, with CP and environmental actors accusing each other of artificially inflating or minimizing burned-area figures, serving the political agenda of their officials. This conflict mainly persisted at the state level, with regional actors stating they got along well with each other, while their superiors were fussing over the numbers.

### External Influences

The description of the different phases already names some external influences, such as the involvement of national and even foreign actors in Phase 2, the pandemic, and political decisions on the federal and state levels that sparked Phase 4. Regarding politics, further external aspects to consider are a perpetual increase in environmental budgets in Mexico at the beginning of the new millennium and then a drastic decrease since 2015: from this year to 2020, Vega (2020) documented an overall reduction of 56% in nominal terms, affecting particularly CONAFOR (minus 66%); CONANP’s budget got reduced by 27%. Numbers provided in the transparency section on CONAFOR’s website (Comisión Nacional Forestal 2025) reveal that between 2020 and 2024, there was a 19.8% recovery in nominal terms. However, given the high accumulated inflation of these years (Instituto Nacional de Estadística y Geografía 2025), this was still a decrease in real terms of 4.5%.

The direct impact of this recent development on FM is unclear from official documents and appears to have had minor effects on personnel in official wildfire brigades in the study area, according to the interviewees. However, following the data provided by CONAFOR’s website, there were direct consequences regarding accompanying programs, such as payments for environmental services (on the national level there was a decrease from 390,000 ha supported by the CONAFOR in 2015 to 177,387 ha in 2023) and temporal work programs that financed prevention activities carried out by local communities. In the interviews, one community representative remarked: “Lately they [fire breaks] got abandoned, no one does it any more“. The total number of maintenances of existing and the establishment of new fire breaks reported by the CONAFOR at the national level in 2015 was 4590 km, and in 2023, only 830 km. Moreover, in recent years the Military sector has undergone considerable strengthening, and a new entity, the National Guard, has been established that also participates in wildfire suppression. Other factors influencing FG include international migration (as challenge to the continuity of personnel in local fire brigades), the deteriorating rule of law in Mexico, and climate change.

### Management Issues

Most management issues are interrelated with external influences and the actors’ objectives and interactions. The following paragraphs are ordered by their perceived importance, based on the number of interviewees who mentioned them. The recent disaggregation of cooperation at the state level and, relatedly, the politicization of FM were the most mentioned issues, leading one firefighter to observe that “the technical part, the operational part, usually do not go well hand in hand with the political part”. The “technical and operational part” refers here to the work of local fire managers and firefighters, while the term “political” in this context referred to personal political agendas of government officials and the rivalry between different secretaries and the related politicians (partisan politics were less critical, as the same party held the federal, state and all municipality governments in the region); another firefighter added “Sometimes it is not so much that people here wouldn’t like to work together, but there are other people at above levels, where there is much jealousy, much dispute”. Related to this, one of the most mentioned problems was the state government’s fire bans, which many other governmental and NGO actors saw as ineffective and counterproductive, reflecting a top-down approach that fails to consider local needs. The lack of cooperation is also related to some actors’ insufficient capacities and know-how, hindering effective fire suppression and putting firefighters at risk. At the same level were the lack of funding for prevention (relating to only temporarily activated brigades) and the loss of cultural fire knowledge.

The loss of fire knowledge was related to emigration, abandonment of agricultural activities, the lack of this topic in formal agricultural training and education (evidenced during the visit to a local agricultural school), and general disinterest among the younger generation. The results are, in some cases, a reduction in agricultural burning, but in other cases, an increasing tendency towards careless fire use. However, one representative of an indigenous community contradicted the official data presented in the paper’s Methodology and the discourse of agricultural burning as the primary fire cause (and not other causes mentioned in some interviews and related mostly to people coming from outside the LSBR, such as hunting, cigarettes along roads, and arson), exclaiming: “There is the problem, well, I have heard this news, that it is the farmers, that it is the producers of corn and beans, who provoke the fires. It is a lie; on this side of the road where we plant corn there are no fires, absolutely, it is on the other side where there are.” To a lesser degree, other community representatives, while not negating the role of agricultural burning, also underlined the importance of other causes and the inappropriateness of placing the blame solely on burning. Also, during the field visits, it was observed that many fires occur along roads used by people passing through the reserve. Markedly, most legal and cultural prevention activities designed top-down by federal and state actors focus almost entirely on agricultural burning.

Whereas most actors, from different actor groups, mentioned these first issues, the following aspects appeared in four interviews each. The first were perverse outcomes of governmental payments for conservation, which are closely related to the loss of cultural traditions mentioned before and, together with budget cuts by the government, have caused a decrease in the maintenance of fire breaks between communities, with one government official stating “today they do not do this anymore, or if they do it, it is because of a governmental program, but not for free, because this custom has been lost, our own governmental system has induced this in the people”. Another issue was the lack of adequate equipment, especially at the community level, including the availability of radios for communicating during the suppression of large fires and the incompatibility of radio equipment of different agencies. Also, four interviewees mentioned dangerous fuel accumulation in areas with effective fire suppression and a lack of prescribed burning, with one interviewee speaking of a “time bomb” regarding potential future megafires. Other aspects mentioned at the same level were the lack of involvement of environmental attorneys, leading to general impunity for arson, and the area’s physical geography, which complicates fire suppression due to steep terrain and frequent strong winds.

Other issues only appeared in three or fewer interviews: the missing participation of agricultural secretaries, increased fire risk due to climate change, the missing spatial fit of prevention activities, security risks for firefighters in areas with presence of criminal groups, a reduction of prevention activities due to the COVID-19 pandemic, lack of coordination and mutual help among local communities, missing dissemination of the NOM015, a lack of renovation of management plans, the lack of more widespread participation of civil society in FM, and the discontinuity of personal, especially at the municipal level (in contrast to field notes from direct observation, which identified this issue as a common problem), expressed in the words of a firefighter: “There can enter very good people but three years pass and there is a change, there is replacement, and we start all over again”.

### Spatial Disparities

Most of the actor roles and management issues presented previously describe the general situation of the study area. However, there are some essential spatial differences. Formal FM activities are much rarer along the Southern border of the LSBR, and actor cooperation is scarce due to the usually lower wildfire incidence in non-fire-prone ecosystems. However, when fires occur, these missing structures along with greater distance from the main firefighting actors hinder suppression efforts. The remainder of the reserve is divided into a western and an eastern part.

The eastern part overall has a history of stronger community participation, continuity of municipal actors, and NGO and university involvement. Together, the actors in this area achieved a considerable lowering of adverse fire impacts in phases 3 and 4. However, in parts of this area, fire suppression, while effective in the short term, has led to dangerous fuel accumulations. In one of the field visits, the authors observed the suppression efforts of a fire in this area that affected more than 2000ha, a rare event in Southern Mexico. Most prescribed burning efforts also occur here, but their extent is far too low. Conflicts between actors in recent years in this area were mediated by prior acquaintance among environmental and CP actors and a generally more integrated approach.

On the contrary, in the western part, conflicts were stronger; later, state CP changed personnel from the eastern to the western part, which eased the situation. At a lower level, there are disparities between communities. Communities in the interior of the LSBR often receive direct forest benefits or obtain payments for environmental services and have received support to set up their fire brigades, making them generally keener to participate in firefighting and forest protection in general, compared to communities at the reserve’s borders. However, even between neighboring communities, there are often important differences in fire use and FM engagement, depending on their prior interactions with other actors and their histories of wildfire impacts.

## Discussion and Conclusions

This paper revises the way multiscale FG plays out in the LSBR, producing an identification of the involved actors, and their roles and interactions, and a diagnosis of local FM issues, which might serve as a guide for future intervention to enhance how fire is managed in the area. However, many of the insights from this effort apply not only to this local case study but also to the revision of FG in other protected areas or regions with important forest resources threatened by changing fire regimes.

The *main findings* of the study include the following: the first premise is to exercise caution when providing generalized answers about FM in a region, even as small as La Sepultura, as there are important intraregional disparities and, in addition, FM dynamics change constantly. This echoes previous studies on FG and the implicated social networks, such as Spies et al. (2018) and Tedim et al. (2020), recognizing the complexity of the wildfire phenomenon and avoiding simplistic solutions. As shown here, a holistic approach that considers different scales, and the input from distinct places within the study area and a diversity of sources, can overcome this tendency.

Similarly, the study underlines the importance, discussed previously by Smith et al. (2021), of not seeing communities and organizations as homogenous blocks, but to interview various representatives of the main actor groups, showing internal differences in opinions and approaches; suffice to add that also these are subject to constant change. The study confirms previous findings regarding different objectives within organizations (Hamilton et al. 2021), various degrees of agreement or opposition of locals toward governmental agencies (Smith et al. 2021), and differences between nearby communities (Ponce-Calderón et al. 2020) and within the same community: between those who own land and those who do not and thus are excluded from decision-making (Martínez-Torres et al. 2018), as well as due to people’s personal experiences (Cammelli et al. 2019). The study did not reveal a specific gender effect on this issue as previously found by Weber et al. (2019), despite the extraordinarily high percentage of male land ownership. Contrary to Bilbao et al (2019), there were no clear findings regarding the influence of age. In addition to these previous findings, the study added that there seems to be an influence of ethnicity, with indigenous groups being less prone to abandoning cultural FM practices. It also showed the importance of contact with external groups shaping local decisions regarding fire use, depending on the intensity of interactions and on the general FM approaches of these external actors.

While being mindful of these complexities, it is important not to lose sight of the bigger picture to ensure a systematic analysis of the main issues that characterize FG in a region. The categorization applied here has shown to be a helpful tool, including the identification of six main themes and the visual representation of actor roles and interactions between different scales, combining visualization approaches from previous PA (Brenner and Vargas 2013) and SNA studies (Kelly et al. 2019).

The most critical *direct implications* of the present work for FG in La Sepultura are discussed in the following lines, dealing with the two main overall governance problems identified here: first, cooperation between actors holding different opinions on FM; and second, community involvement and government-community interactions, reflecting the main objectives of FM outlined by Bennett and Satterfield (2018) which revolve around actor interactions and also underline the importance of ample and fair stakeholder participation.

Disagreements regarding the optimal way for FM are a common feature of regions with a high incidence of wildfire, both in the industrial world and in developing countries (Bacciu et al. 2022; Devisscher et al. 2018; Fischer and Jasny 2017; Neger et al. 2024a; Spies et al. 2018; Spencer 2023; Tedim et al. 2020). This includes differing opinions on fuel management, technical and cultural fire use, and, as was apparent in the present example, on the organization of fire suppression and the documentation of wildfire impact data. Adding to the findings of previous studies regarding social networks in FM (Faas et al. 2019; Kelly et al. 2019; Spencer 2023), the study evidenced that these disagreements or even conflicts are an obstacle to effective FM and can lead to worsened fire impacts, for instance, due to the lack of more widespread prescribed burning because of the resistance of some actors. Moreover, it creates security risks for firefighters. Different approaches in this case study were mainly related to the general focus of organizations; scale and jurisdictional limits seem to have had less influence as in other cases (Fleming et al. 2015; Hamilton et al. 2023; Kelly et al. 2019), although there was a tendency of regional actors to be more prone to learning and cooperation than at the more politicized state level.

While showing these negative impacts of lack of coordination in FM, the fieldwork for this research also revealed some hints on how to diminish or overcome them, considering that the actors share the same overall goal, which is a prerequisite for any successful coordination in EG (Larson et al., 2018), in this case of optimizing FM in the area. First, it documented that as conflicts are related to differences in understanding and experience, they can change over time, through learning processes. Also, since conflicts are not personal but technical, there is a potential to gradually build trust. The importance of interpersonal relationships, and of the flexible application of policies (such as how the NOM-015 is implemented) reflect earlier findings in other fire and general risk management contexts by studies which put the issue of trust at the centre of their analysis (Olsen and Sharp 2013) and point to its fundamental role for successful agency, preceding any technical enhancements (Earle 2010). In the present case, the intervention in FM activities within the reserve’s boundaries by CONANP, which adopted a relatively neutral role apart from the struggles between other governmental agencies, might have contributed to this trust-building process; also, the NGO involvement in the eastern part of the LSBR might have played a role in the more fluent cooperation in this area. What is clear from the present example is that these processes take time and are obstructed by constant rotation of personnel related to political cycles, as documented also by Devisscher et al. (2018), and by abrupt changes in governance structures.

The need for governmental agencies to communicate and reach agreements is also necessary to avoid confusion in their interactions with local communities. Again, long-term involvement of government officials fosters trust and learning processes, leading, for instance, to the abandonment of unproductive punitive measures against agricultural burning accidents. In the best case, governmental agencies and local communities see each other as allies addressing a common problem, as communities themselves are the first to be affected by negative wildfire outcomes (Carmenta et al. 2021). Respect for local organization is key, together with the avoidance of one-size-fits-all measures, and an approach based on a profound understanding of the specific problematic of each community, considering different opinions held by locals and supporting grassroots initiatives (see Larson et al. 2018).

The initial impression from phase 5 indicated an improvement over phase 4 in coordination, though it remained less consolidated than in phase 3. Government-community relationships enhanced considerably during phases 2 and 3, but since then, this integration has stagnated. The extremely high fire incidence in 2023 will likely lead to reflection and shake up the strategies of different actors. In the best-case scenario, this could lead to overcoming the obstacles to effective cooperation, making the need for IFM clear to everyone, and focusing on the common overall goal of risk reduction and prevention. It could also spark the realization of the importance of preparedness at the local level, empowering community efforts. However, the opposite might also be true, as politicians leading fire-related institutions might be lured to revert to restrictive policies and suppression-only approaches in response to rising fire numbers.

*Future research needs* identified by this study relate especially to two aspects that were seen in ambiguous ways and for which no clear solution seemed to be available. One of them was the perverse outcome of payments for conservation, a bane of environmental conservation efforts in many areas and often related to neoliberal environmental politics and the commodification of nature, with the vertical application of subsidies that accustom locals to conserve their natural resources due to a monetary interest, eroding traditional communal conservation practices (Shapiro-Garza 2019). However, community representatives also defended the need to obtain compensation for FM efforts, highlighting their benefits beyond the community and the difficulties and risks involved in this work. In this regard, particularly in the context of reduced environmental conservation budgets, it is necessary to study and test innovative solutions that support local efforts in a just way. A potential solution might be found in the apparently higher commitment of communities obtaining direct forest benefits, as shown here and elsewhere (Carmenta et al. 2021).

Another aspect without a clear result was the role of agricultural burning, in line with other studies that question the generalized blaming of cultural FM (Bilbao et al. 2019; Ponce-Calderón et al. 2020). Official statistics identify agricultural burning as the probable cause of most wildfires in the area. This supposition was voiced in many interviews with government officials, even those who lean towards IFM, contrasted by statements at the local level and observations in field. A clearer understanding is crucial, as most governmental resources for cultural and legal prevention revolve around better control measures for agricultural burning, potentially misaligned with the real fire problems, at least in some places.

In addition to the need for further research on these issues, payments for conservation efforts and the role of agricultural burning, further research is necessary in the LSBR to determine the reasons behind the soaring fire numbers in recent years. The mentioned management problems might be an important factor in increased fuel accumulation and fire spread; however, they are embedded in what is identified here as external influences, particularly public policies affecting rural communities, social phenomena such as migration, and climate change. In terms of the evaluation of effective EG (Bennett and Satterfield 2018), it would be necessary to apply an interdisciplinary research effort among social scientists, fire ecologists, meteorologists, amongst others, to single out the outcomes of specific aspects and to apply detailed counterfactual analysis, to determine the real impact of the area’s FG structure and dynamics, a proposition beyond the means of the present study that was entirely based on social sciences methods.

Moreover, a more profound analysis from a PE perspective regarding power asymmetries and structural inequalities (Robbins 2012), taking as a reference the hierarchies of actor involvement in decision-making shown in the figures, could yield additional insights. Also, the dynamic nature of governance shown here, in line with previous research in other areas (Smith 2021), means that research in this context needs to be an ongoing effort. The present study was limited in this regard, as it had access to past developments only through the revision of previous publications and the memories of the interviewees, but no direct observation, apart from the years 2021 to 2023. A longitudinal approach would be beneficial for enhancing and deepening understanding of FG, an objective that is difficult to achieve in the context of short-lived projects and short-term productivity goals in contemporary academic practice.

## Data Availability

Relevant data are provided within the manuscript. We do not provide further information from interview data in a public repository, to ensure the anonymity of the persons who were interviewed for this research. On request, we can make edited, non-sensitive data available to interested reviewers or readers.
